# Newly Diagnosed Autoimmune Type 1 Diabetes Presenting With Severe Diabetic Ketoacidosis in a 92-Year-Old Woman: A Rare Diagnosis in the Tenth Decade of Life

**DOI:** 10.7759/cureus.119616

**Published:** 2026-11-13

**Authors:** Fatima Saleem Mohd Saleem, Hamza Hamid, Jerome Iyamu, Syed Ali Oraizi Jafery

**Affiliations:** 1 General Medicine, Princess Alexandra Hospital, Harlow, GBR; 2 Diabetes and Endocrinology, Princess Alexandra Hospital, Harlow, GBR

**Keywords:** diabetes mellitus in elderly, diabetic ketoacidosis, hyperglycaemia, tenth decade of life, type 1 diabetes mellitus (t1dm)

## Abstract

New-onset type 1 diabetes mellitus is rare in very elderly patients. We describe a 92-year-old woman with no previous diagnosis of diabetes who presented with a one-week history of polyuria, polydipsia, generalized malaise, diarrhea, and vomiting, alongside a one-month history of unintentional weight loss and reduced appetite. On admission, she was found to be in severe diabetic ketoacidosis (DKA), meeting Joint British Diabetes Societies severity criteria on the basis of blood ketones reported as "HI" (exceeding the >6 mmol/L threshold); arterial pH was 7.24, bicarbonate 19.2 mmol/L, and lactate 3.56 mmol/L. Blood glucose was above the measurable range, also reported as "HI." Contrast-enhanced computed tomography (CT) of the abdomen and pelvis was later performed to exclude an underlying secondary cause of hyperglycemia, including pancreatic pathology or malignancy, and revealed no significant abnormalities. Positive anti-glutamic acid decarboxylase (anti-GAD) antibodies and a low C-peptide level supported a diagnosis of autoimmune, insulin-deficient type 1 diabetes. She was managed according to DKA protocol, and following resolution of DKA, she was discharged with diabetes and primary care follow-up. This case highlights the diagnostic value of autoimmune antibody testing in elderly patients presenting with severe DKA, given that age-based assumptions about diabetes subtype may otherwise lead to misclassification and inappropriate management.

## Introduction

Globally, an estimated 9.5 million people live with type 1 diabetes mellitus (T1DM) [1]. T1DM is typically diagnosed in the younger age group; however, clinical observation suggests that there is an increasing frequency of diagnosis in the elderly population [2].

Identification of adult-onset T1DM is important because misrecognition as type 2 diabetes mellitus (T2DM) may lead to inappropriate management and hence morbidity [3]. Autoimmune diabetes presenting for the first time as diabetic ketoacidosis (DKA) in the tenth decade of life is rare. DKA arising at this age should prompt a search for a precipitant or secondary cause of new hyperglycemia.

## Case presentation

A 92-year-old woman was brought to hospital by ambulance following several days of diarrhea, vomiting, and inability to tolerate oral intake. Before admission, she was independent, lived with her daughter, and was her daughter's main carer. Her family reported a 5 kg unintentional weight loss over the past month. During the same period, she had also experienced polyuria, polydipsia, and progressively worsening fatigue. She had also developed oral thrush several days before admission, for which her general practitioner (GP) had prescribed nystatin.

There was no prior history of diabetes mellitus, nor any family history of the condition. Her background medical history included hypertension, congestive cardiac failure, and hypothyroidism. Her regular medications were ramipril 10 mg once daily, spironolactone 25 mg once daily, levothyroxine 75 μg once daily, nebivolol 1.25 mg once daily, atorvastatin 40 mg, aspirin 75 mg once daily, and lubricating eye drops. She had no known drug allergies.

On ambulance assessment, she was noted to be unwell and clammy, with high capillary blood glucose. Initial observations showed heart rate (HR) 67/minute, blood pressure (BP) 113/57 mmHg, SpO₂ 97% on room air, and respiratory rate 38/minute. Her Glasgow Coma Scale (GCS) was 14/15 (E4 V4 M6) [4].

On arrival at the hospital, direct history was limited by delirium, and collateral history was obtained from family members. Initial arterial blood gas (ABG) analysis (obtained via arterial puncture on room air) demonstrated a pH of 7.24, bicarbonate 19.2 mmol/L, lactate 3.56 mmol/L, ketones measured as "HI" (above the 7 mmol/L analytical range of the point-of-care device), and glucose above the measurable range of the point-of-care device. According to the Joint British Diabetes Societies (JBDS) severity criteria, the markedly elevated blood ketone level was consistent with severe DKA [5]. She had acute kidney injury (AKI) stage 3, alongside hyperkalemia of 6.1 mmol/L. Inflammatory markers were elevated on admission, and chest radiography was consistent with a lower respiratory tract infection. Initial blood investigations and subsequent autoimmune testing are summarized in Table 1.

**Table 1:** Blood investigations on admission and autoimmune markers suggestive of type 1 diabetes. ABG: arterial blood gas

Investigation	Result	Normal value
pH (initial ABG)	7.24	7.35 to 7.45
Bicarbonate	19.2 mmol/L	22 to 26 mmol/L
Lactate	3.56 mmol/L	0.5 to 1.6 mmol/L
Ketones	High, above measurable range	<0.6 mmol/L
Glucose	High, above measurable range	4.0 to 7.8 mmol/L
Creatinine	151 μmol/L	53-97 μmol/L
Estimated glomerular filtration rate	28 mL/min/1.73 m²	60-150 mL/min/1.73 m²
Potassium	6.1 mmol/L	3.5-5.3 mmol/L
White blood cells	13.7 x 10⁹/L	4.0-11.0 x 10⁹/L
C-reactive protein	27 mg/L	<5 mg/L
Procalcitonin (PCT)	0.1 ng/mL	0.0-0.5 ng/mL
HbA1c	153 mmol/mol	20-42 mmol/mol
Troponin I trend	144.7 → 132.4 ng/L	0.0-1.9 ng/L
Thyroid-stimulating hormone	1.53 mU/L	0.35-5.00 mU/L
Anti-glutamic acid decarboxylase (anti-GAD) antibodies	83.80 U/mL	<1.00 U/mL
Zinc transporter 8 antibodies	<10.0 U/mL	<15 U/mL
IA-2 (islet antigen 2) antibodies	<10.0 U/mL	<10.0 U/mL
C-peptide	162 pmol/L	258-1,718 pmol/L

She was managed for DKA in accordance with local trust guidelines, with intravenous antibiotics commenced for the concurrent infection. Her DKA resolved uneventfully, with no clinical signs of cerebral edema or other treatment-related complications. Admission HbA1c was 153 mmol/mol. ECG changes were noted during admission (Figure 1), along with a raised, non-dynamic troponin (144.7 → 132.4; normal value 0.0 to 1.9 ng/L) without chest pain, considered a likely type 2 myocardial infarction.

**Figure 1:**
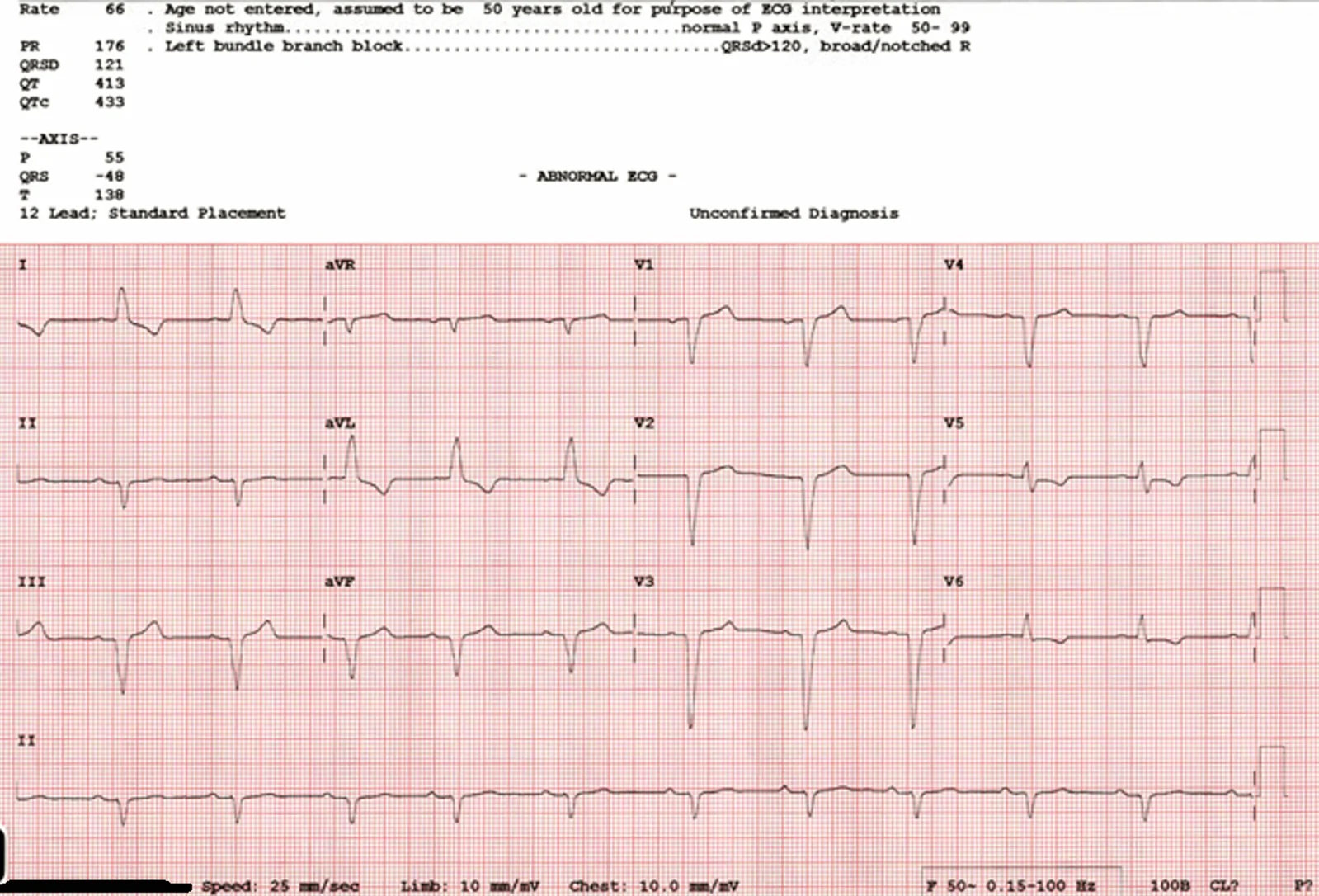
ECG at admission demonstrated sinus rhythm with left axis deviation, incomplete left bundle branch block, and a ventricular rate of 66 beats per minute.

Given the atypical age at presentation, further investigations were recommended to establish the underlying etiology of her diabetes, including computed tomography (CT) imaging (Figure 2) to exclude occult pancreatic malignancy, pancreatic enzyme measurement, autoimmune antibody testing, C-peptide with paired glucose, and thyroid function testing, which showed a thyroid-stimulating hormone (TSH) level of 1.53 mU/L.

**Figure 2:**
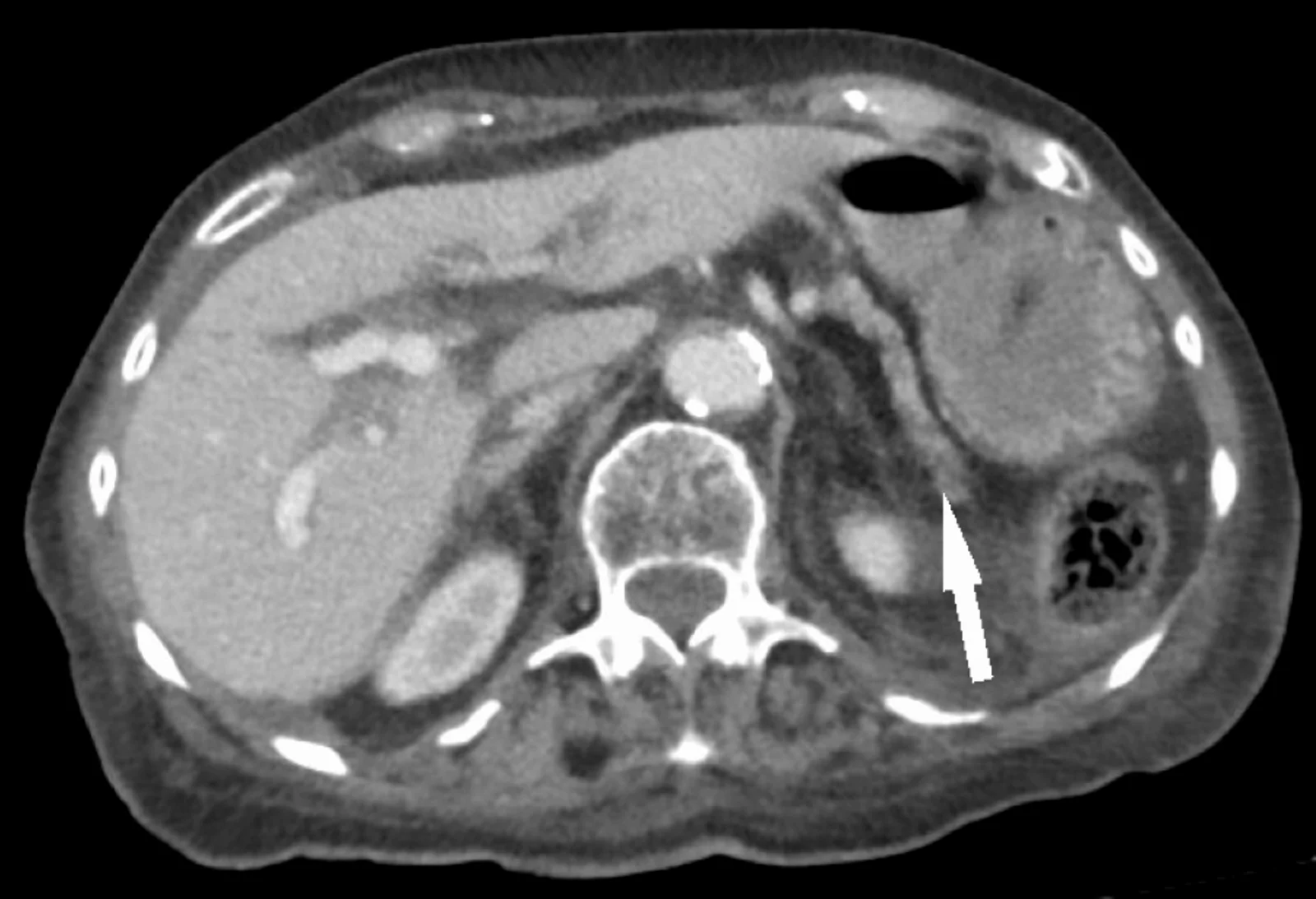
Computed tomography (CT) abdomen and pelvis with contrast showing mild diffuse atrophy of the pancreas with no obvious focal lesion (arrow). The peripancreatic fat planes are clear, and there is no collection. The radiologist advised that atrophy of the pancreas is age-related.

CT of the abdomen and pelvis showed no radiological evidence of pancreatitis or pancreatic malignancy. An incidental finding of mild right hydronephrosis with a possible ureteric calculus prompted urological review, with conservative outpatient follow-up arranged, including repeat blood tests and CT kidneys, ureters, and bladder imaging post-discharge.

Subsequent results confirmed positive anti-glutamic acid decarboxylase (anti-GAD) antibodies (83.80 U/mL) and a low C-peptide level (162 pmol/L, paired with high glucose), with zinc transporter 8 antibodies negative (<10.0) and IA-2 (islet antigen 2) antibodies negative (<10.0), establishing a diagnosis of autoimmune T1DM. She was discharged on insulin glargine six units once daily, provided with a continuous glucose monitoring system, and arranged appropriate diabetes follow-up.

## Discussion

T1DM is classically a disease of the young; however, autoimmune β-cell destruction may occur at any age [6]. There is increasing recognition of adult-onset autoimmune diabetes, but diagnosis in the ninth or tenth decades of life is uncommon [7]. This report emphasizes the importance of maintaining vigilance for autoimmune diabetes even in the very elderly.

New-onset diabetes in older adults commonly represents T2DM. Symptoms such as weight loss, polyuria, polydipsia, and fatigue may be attributed to age-related frailty, current illness, or progressive T2DM [8]. In elderly individuals presenting with DKA, clinicians may have secondary causes of severe hyperglycemia, including infection, pancreatic disease, medication-related hyperglycemia, or previously undiagnosed T2DM with ketosis-prone features, higher on the differentials. In this patient, the extreme age at presentation prompted a comprehensive evaluation for alternative etiologies, including pancreatic malignancy and pancreatitis.

Latent autoimmune diabetes in adults (LADA) represents the most frequent form of autoimmune diabetes diagnosed later in life [9]. According to established diagnostic criteria, LADA is defined by adult onset, islet autoantibody positivity, and preserved endogenous insulin secretion, allowing patients to avoid ketoacidosis and remain insulin independent for at least six months following diagnosis [10]. In contrast, our patient presented with severe hyperglycemia, significant ketosis, and a low C-peptide level, indicating advanced β-cell failure more in keeping with classic autoimmune type 1 diabetes than LADA [11,12]. The presence of anti-GAD antibodies is the most common immunological marker of autoimmune diabetes in adults [13].

Maturity-onset diabetes of the young (MODY) was also excluded given her presentation in the tenth decade of life, absence of an autosomal dominant family history, severe ketoacidosis, and positive anti-GAD antibodies. Severe DKA occurring as the first presentation of autoimmune diabetes in adults in the tenth decade of life is extremely rare [7]. The initial presentation may be complicated by delirium, creating diagnostic difficulties and potentially delaying recognition of the underlying disorder.

Autoimmune thyroid disease is the most frequently associated autoimmune condition in individuals with T1DM [14]. The patient had a pre-existing diagnosis of hypothyroidism of 30 years' duration, which raises the possibility of an underlying autoimmune process.

The increasing incidence of diabetes in older adults necessitates careful differentiation between T1DM and T2DM [9]. Autoimmune diabetes in adults is frequently not identified and is commonly treated as type 2 diabetes, which can lead to inappropriate or delayed treatment [15,16]. Autoimmune diabetes should be suspected in adults of any age who present with hyperglycemia and features such as rapid weight loss or ketosis, since age and BMI alone should not be used to exclude it [17]. In such cases, measurement of islet autoantibodies and C-peptide levels should be considered [11].

## Conclusions

Autoimmune type 1 diabetes can present for the first time even in the tenth decade of life. This case demonstrates that advanced age should not exclude T1DM from the differential diagnosis of severe hyperglycemia or DKA. Autoantibody testing and C-peptide measurement are valuable diagnostic indicators when the clinical presentation is atypical. Early recognition of autoimmune diabetes allows for prompt initiation of insulin therapy and may prevent significant complications associated with delayed diagnosis.

## References

[1] Ogle GD, Wang F, Haynes A (2025). Global type 1 diabetes prevalence, incidence, and mortality estimates 2025: results from the International Diabetes Federation Atlas, 11th Edition, and the T1D Index Version 3.0. Diabetes Res Clin Pract.

[2] LeRoith D, Biessels GJ, Braithwaite SS (2019). Treatment of diabetes in older adults: an Endocrine Society* clinical practice guideline. J Clin Endocrinol Metab.

[3] Maahs DM, West NA, Lawrence JM, Mayer-Davis EJ (2010). Epidemiology of type 1 diabetes. Endocrinol Metab Clin North Am.

[4] Teasdale G, Maas A, Lecky F, Manley G, Stocchetti N, Murray G (2014). The Glasgow Coma Scale at 40 years: standing the test of time. Lancet Neurol.

[5] (2026). Joint British Diabetes Societies for Inpatient Care. The management of diabetic ketoacidosis in adults. Revised March.

[6] Leslie RD, Williams R, Pozzilli P (2006). Clinical review: type 1 diabetes and latent autoimmune diabetes in adults: one end of the rainbow. J Clin Endocrinol Metab.

[7] Ahmad W, Bates C, Dale L, Siddaramaiah N (2022). Newly diagnosed type 1 diabetes in a 93 year old. BMJ Case Rep.

[8] Bigelow A, Freeland B (2017). Type 2 diabetes care in the elderly. J Nurse Pract.

[9] Tomic D, Harding JL, Jenkins AJ, Shaw JE, Magliano DJ (2025). The epidemiology of type 1 diabetes mellitus in older adults. Nat Rev Endocrinol.

[10] Lundholm MD, Zhou K (2025). Latent autoimmune diabetes in adults: not type 1, not type 2, a little of both. Cleve Clin J Med.

[11] Leighton E, Sainsbury CA, Jones GC (2017). A practical review of C-peptide testing in diabetes. Diabetes Ther.

[12] Fourlanos S, Dotta F, Greenbaum CJ, Palmer JP, Rolandsson O, Colman PG, Harrison LC (2005). Latent autoimmune diabetes in adults (LADA) should be less latent. Diabetologia.

[13] Sanyal D (2020). Current perspective on auto-antibodies in type 1 diabetes. Indian J Endocrinol Metab.

[14] Kawasaki E (2014). Type 1 diabetes and autoimmunity. Clin Pediatr Endocrinol.

[15] Thomas NJ, Lynam AL, Hill AV (2019). Type 1 diabetes defined by severe insulin deficiency occurs after 30 years of age and is commonly treated as type 2 diabetes. Diabetologia.

[16] Shidler KL, Letourneau LR, Novak LM (2020). Uncommon presentations of diabetes: zebras in the herd. Clin Diabetes.

[17] (2026). National Institute for Health and Care Excellence. Type 1 diabetes in adults: diagnosis and management (NG17). https://www.nice.org.uk/guidance/ng17.

